# Preeclampsia: a bioinformatics approach through protein-protein interaction networks analysis

**DOI:** 10.1186/1752-0509-6-97

**Published:** 2012-08-08

**Authors:** Eduardo Tejera, João Bernardes, Irene Rebelo

**Affiliations:** 1 Department of Biological Sciences, Biochemistry, Faculty of Pharmacy, University of Porto, Portugal/Institute for Molecular and Cell Biology (IBMC), Porto, Portugal; 2 Department of Obstetrics and Gynecology, Faculty of Medicine, University of Porto, Department of Obstetrics and Gynecology, Sao Joao Hospital./INEB – Institute of Biomedical Engineering, University of Porto, Porto, Portugal

## Abstract

**Background:**

In this study we explored preeclampsia through a bioinformatics approach. We create a comprehensive genes/proteins dataset by the analysis of both public proteomic data and text mining of public scientific literature. From this dataset the associated protein-protein interaction network has been obtained. Several indexes of centrality have been explored for hubs detection as well as the enrichment statistical analysis of metabolic pathway and disease.

**Results:**

We confirmed the well known relationship between preeclampsia and cardiovascular diseases but also identified statistically significant relationships with respect to cancer and aging. Moreover, significant metabolic pathways such as apoptosis, cancer and cytokine-cytokine receptor interaction have also been identified by enrichment analysis. We obtained FLT1, VEGFA, FN1, F2 and PGF genes with the highest scores by hubs analysis; however, we also found other genes as PDIA3, LYN, SH2B2 and NDRG1 with high scores.

**Conclusions:**

The applied methodology not only led to the identification of well known genes related to preeclampsia but also to propose new candidates poorly explored or completely unknown in the pathogenesis of preeclampsia, which eventually need to be validated experimentally. Moreover, new possible connections were detected between preeclampsia and other diseases that could open new areas of research. More must be done in this area to resolve the identification of unknown interactions of proteins/genes and also for a better integration of metabolic pathways and diseases.

## Background

Preeclampsia is a pregnancy related disease associated with hypertension, proteinuria and increased maternal and perinatal morbidity and mortality, without known underlying mechanism and preventive treatment [1,2]. On the other hand, the future health or possible risks of women with previous history of preeclampsia are important areas of investigation. In this direction, it is well known the increased risk of future cardiovascular disease and renal dysfunction, however, other risks are also being discussed [1,3-5]. Owing to the morbidity and mortality of this pregnancy related disease and the possible multifactorial causes involved [1-5], several experimental procedures have been applied by researchers in the last two decades, evidently, generating an elevated number of unprocessed information.

Although some bioinformatic analysis has been performed in particular microarray assays [6,7], an extensive data evaluation and processing has not yet been performed. Furthermore, the capabilities of bioinformatics tools for gene prioritization, network analysis, gene ontology and gene-disease relationships [8,9], together with all available data on protein/gene expression during preeclampsia bring an interesting and valuable opportunity for an *in-silico* study of the disease. Therefore, the present study is focused on two main areas: I) collection and basic analysis of the genes/proteins-diseases dataset, including, protein-protein interaction network and pathway enrichment analysis and II) exploration of the related gene-diseases in order to evaluate other genetic diseases possibly related with preeclampsia.

## Results

### Protein-protein interaction network analysis

**Figure 1:**
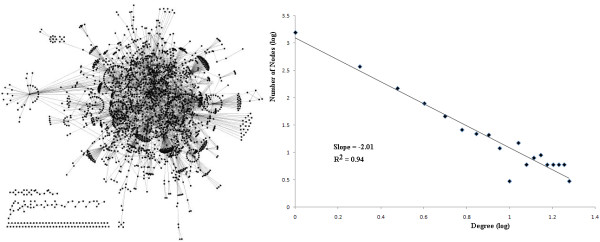
**PPI network and topology. Left)** PPI network and **Right)** Degree distribution. The degree distribution follows a power law distribution.

Preeclampsia PPI network topology reveals (Figure 1) a similar behavior with respect to general topology of PPI following a power law behavior [10] and therefore scale-free properties. These types of networks have the particular feature that some nodes are highly connected compared with others on the same network. These highly connected nodes (hubs) in general, represent important proteins/genes in biological terms and therefore are treated with special attention.

The top 50 genes with high scores and also present in the initial set (347) are shown in Table 1, however, other genes were found with high scores value but there are not part of the initial gene group. As expected some of the selected genes like FN1, FLT1, F2, VEGFA, PGF, TNF, NOS and INHBA, are well known preeclampsia relates genes (see discussion) and several of them are related with signaling pathways.

**Table 1:** Top 50 genes obtained by analysis of the PPI network

**Gene symbol**	**Entrez**^**1**^	**Score I**	**Gene symbol**	**Entrez**^**1**^	**Score I**
4067	LYN	983.5136	2324	FLT4	426.74
2335	FN1	823.9037	6767	ST13	425.5747
29110	TBK1	672.1386	873	CBR1	424.257
8826	IQGAP1	635.9629	667	DST	414.0964
1759	DNM1	628.7167	9495	AKAP5	412.7567
5573	PRKAR1A	622.5864	8601	RGS20	408.6292
10397	NDRG1	590.672	2896	GRN	401.0426
2923	PDIA3	559.3358	4323	MMP14	400.1593
5339	PLEC	541.5909	55697	VAC14	394.4482
6303	SAT1	515.6939	5228	PGF	393.3167
2321	FLT1	508.3947	8553	BHLHE40	392.8039
2317	FLNB	505.1671	604	BCL6	385.7928
4221	MEN1	499.019	5054	SERPINE1	382.5547
7067	THRA	487.6884	4597	MVD	380.0219
2147	F2	482.7173	5569	PKIA	377.6185
3959	LGALS3BP	475.7565	140885	SIRPA	375.9565
10603	SH2B2	473.9241	3624	INHBA	367.5795
7422	VEGFA	465.5212	10272	FSTL3	367.2976
8815	BANF1	461.918	4846	NOS3	361.5554
23650	TRIM29	448.6764	3553	IL1B	357.2569
7124	TNF	441.7007	3880	KRT19	355.4412
904	CCNT1	440.8304	9444	QKI	354.3211
3556	IL1RAP	430.4483	8773	SNAP23	348.0265
9131	AIFM1	429.2521	27250	PDCD4	345.9363
6449	SGTA	429.1609	5291	PIK3CB	345.5114

The table shown the first 50 genes selected according to the score values and arranged in descendent order. We also provide the gene symbol the associated Entrez Gene identifier.

Notes: 1) Entrez gene ID.

**Figure 2:**
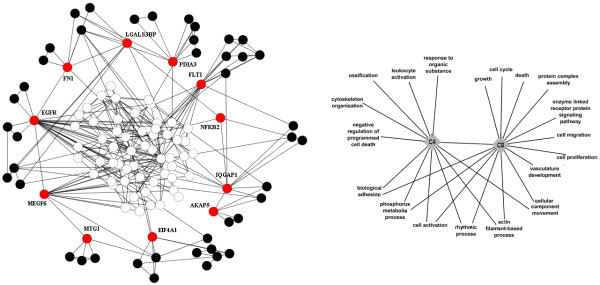
**Communality and clusters analysis. Left)** Representation of the largest connected community. Red nodes represent the genes involved in communities overlapping. White nodes represent the bigger community. **Right)** Representation of C8 and C4 clusters and the related statistically significant biological process obtained by gene ontology enrichment analysis.

A total of 27 communities (k = 3) covering 161 genes were identified by communality analysis. In Figure 2 (Left) we represent those communities that are superimposed to form a large connected graph. The genes involved in communities overlapping are also highly represented in the Table 1 (and also the genes members of the large community). The model based clustering analysis reveal an optimal number of 8 clusters (BIC = 152192.4) with an ellipsoidal distribution with equal volume, shape and variable orientation. The genes are grouped in the clusters (C1…8) as follow: C1 (67), C2 (56), C3 (1806), C4 (59), C5 (133), C6 (23), C7 (95) and C8 (161). The C8 and C4 correspond with the highest mean scoring value: 393.3 and 348.9 respectively, and contain all the 100 genes with highest score values (part or not of the initial gene set). Furthermore, 161 genes of C8 are also the same genes detected in the communality analysis.

Gene ontology (GO) enrichment analyses were performed in all obtained clusters. However, for simplicity only C4 and C8 are presented (Figure 2 Right). The GO analysis reveals that C8 comprise several processes related with angiogenesis, apoptosis and cell proliferation and also shared with C4 several processess involved in cell activation and biological adhesion. The relation between these processes as well as the fact that both groups are representative of the highest scored genes could indicate a particular relevance of the clusters in terms of genes-disease relationship. On the other hand, also these processes are well known involved in preeclampsia and are also consistent with the pathway enrichment analysis.

### Diseases and metabolic pathway enrichment analysis

Several types of diseases were found statistically significant in the enrichment analysis; partial results are presented in Table 2. Obviously, preeclampsia and even hypertension have to be present in the analysis. In the GAD database there are several disease classes and beside the presented in Table 2, others like hematologic (p-value = 6.1E-06) and renal diseases (p-value = 2.5E-04) were also significant. Even when we present only the results obtained with GAD database, analysis was also performed with OMIN database confirming the ovarian cancer (p-value = 0.019) and also indicating colorectal cancer as statistically significant (p-value = 0.011), however, better results were obtained with GAD and also more consistent with literature information and pathway enrichment analysis.

**Table 2:** The diseases enrichment analysis

**Disease classes**	**Disease**	**p-value**
Cancer (p-value = 1.24E-11)	breast cancer	9.76E-07
	ovarian cancer	4.30E-04
	lupus erythematosus	4.4E-02
Cardiovascular (p-value = 4.28E-9)	myocardial infarction	8.7E-09
	coronary artery disease	7.38E-06
	Stroke	6.16E-05
	heart disease, ischemic	9.7E-03
	blood pressure, arterial	8.2E-03
	inflammatory bowel disease	2.9E-02
Aging (p-value = 5.23E-05)	Alzheimer's Disease	2.36E-07
	Longevity	2.17E-04
	atherosclerosis	3.7E-02
	Arthritis	3.60E-02
Reproduction (p-value = 7.9E-03)	preeclampsia	1.29E-05
	pregnancy loss, recurrent	6.08E-04

The table provides the p-value obtained from disease enrichment analysis according to the Disease Class and also the sub-classes diseases following the GAD database structure. The results showed are partials as discussed in the respective section.

It is important to consider that several genes in the PPI network do not present a known relation with specific diseases, at least reported in the GAD or OMIN databases. Only around 30% of the 2400 genes were found in the databases. This difficulty means that we have to be cautious with the preeclampsia genes-diseases relationships and with the reliability of the statistical p-value, even when some important and significant inferences, can be made.

A similar situation occurred with the pathway enrichment analysis (Table 3). Even when the KEGG database is the most representative of our gene space and high coherence was noticed with the physiopathology of PE, the results only cover around 50% of the initial 2400 genes. A similar procedure was also performed with the Reactome and BioCarta databases with a less covering (37% and 27% respectively) and showing a high coherence with Table 3 results. These databases reveal other significant pathways like NGF, PDGF, BMP, EPO and EGFR signaling as well as apoptosis and hemostasis pathways (data not shown). Some cancerous pathways (i.e. prostatic, pancreatic and lung) were also found statistically significant in KEGG but were excluded from Table 3, in order to simplify, because many of them have similar reactions with the general cancer pathway, already presented in the Table 3.

**Table 3:** The KEGG pathway enrichment analysis

**Pathway description**	**p-value**	**Pathway description**	**p-value**
Pathways in cancer	1.10E-42	Fc gamma R-mediated phagocytosis	2.90E-07
Focal adhesion	3.50E-29	RIG-I-like receptor signaling pathway	5.30E-07
Apoptosis	2.10E-17	Chemokine signaling pathway	5.90E-07
Neurotrophin signaling pathway	3.40E-17	Leukocyte transendothelial migration	6.20E-07
TGF-beta signaling pathway	5.10E-16	VEGF signaling pathway	8.60E-07
T cell receptor signaling pathway	8.90E-15	NOD-like receptor signaling pathway	1.20E-06
ErbB signaling pathway	1.80E-14	mTOR signaling pathway	7.00E-06
B cell receptor signaling pathway	8.70E-14	Complement and coagulation cascades	7.90E-06
Adherens junction	2.00E-12	Tight junction	1.10E-05
Fc epsilon RI signaling pathway	3.60E-12	RNA polymerase	1.60E-05
ECM-receptor interaction	6.80E-12	Ubiquitin mediated proteolysis	2.20E-05
Insulin signaling pathway	3.00E-10	GnRH signaling pathway	1.10E-04
Regulation of actin cytoskeleton	3.10E-09	Hematopoietic cell lineage	1.20E-04
Toll-like receptor signaling pathway	1.01E-08	Cytokine-cytokine receptor interaction	2.00E-04
Wnt signaling pathway	1.10E-08	Hedgehog signaling pathway	3.70E-04
Cell cycle	1.50E-08	Natural killer cell mediated cytotoxicity	3.80E-04
MAPK signaling pathway	1.50E-08	Cytosolic DNA-sensing pathway	6.70E-04
Adipocytokine signaling pathway	7.50E-08	Notch signaling pathway	2.60E-03
Progesterone-mediated oocyte maturation	2.50E-07	Renin-angiotensin system	1.80E-02

The table provides the p-value obtained from pathway enrichment analysis in the KEGG database. The pathways names correspond with the KEGG nomenclature. The results showed are partials as discussed in the respective section.

**Figure 3:**
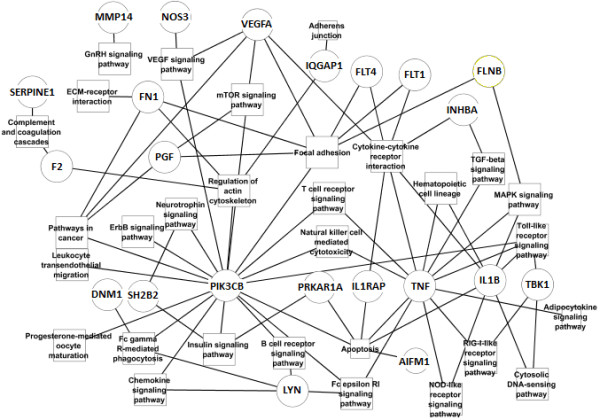
**Genes-metabolic pathways interaction.** The genes and pathways were selected after hubs detection (see Table 1) and enrichment analysis respectively (see Table 3).

In order to simplify and enhance the understanding of the involved pathways and their relationship with the selected hubs, a fusion between both was made (Figure 3). However, it is important to exalt that from the 50 hubs previously selected; only 22 present some significant pathway association with Table 2. The genes: NDRG1, LGALS3BP, BANF1, SGTA, TRIM29, RGS20, PLEC, GRN, ST13, AKAP5, FSTL3, DST, PKIA, QKI, MLF2 and KRT19, for example, were not found in the KEGG database.

## Discussion

### PPI analysis

The top 50 genes selected were manually analyzed with the scientific literature in order to validate its connection with PE or even changes during pregnancy and we corroborate that several of them like FLT1, FLT4, VEGFA, PGF, TNF, FN1, F2 and NOS3 can be related to the main lines of research in preeclampsia pathogenesis hypothesis and specially angiogenesis [11-16]. Moreover, PGF, INHBA and related inhibin as well as activin proteins have been considered in several predictive model of PE [17,18]. The presence of those genes in our selection could validate the method applied and increase our confidence with respect to those genes that are poorly explored or unknown in their association with preeclampsia. In the latter group, we have for example the genes LYN, PDIA3, NDRG1 and TBK1. The PDIA3 genes encode an endoplasmic protein highly related with folding processes and in its relation with pregnancy only one article was found [19] revealing that PDIA3 gene intervene in trophoblast invasion via interleukin (IL) 11. Similarly, TBK1 could also intervene during the first moments of pregnancy to secure the implantation in relation with the nuclear factor kappa B [20]. However, there are not articles of PDIA3, TBK1 or LYN related with preeclampsia. Moreover, only one article was found describing an increased expression of NDRG1 during preeclampsia [21]. A similar problem is found with other genes such as IQGAP1, DNM1, SAT1, MEN1 and SH2B2 that also have been little explored during pregnancy.

Cluster analysis indicates that mainly C8 but also C4 probably embrace the most significant genes, at least related with centrality. All genes highest scored are part of these clusters as well as the large community graph and as we can notice the genes that lead to a community superposition are also highly scored in Table 1 (Figure 2). On the other hand there are other genes like ENG, VEGFB and INHA that are well known related to preeclampsia and are also part of the C8 cluster [11-18]. It is important to notice that there are other genes not shown in Table 1 because were not present in the initial gene set. In this group, EGFR and GRB2, are both with highest scores and there are both well related with preeclampsia [22,23]. Thus, even when the work presented focused on the analysis of the initial group, it is possible that relevant genes were identified by PPI topology but not included in the initial subset. Moreover, the GO enrichment analysis clearly reveals that C8 and C4 clusters are related with angiogenesis, apoptosis and biological adhesion, which are also biological processes obtained with the pathways enrichment analysis. Angiogenesis and related processes (i.e. vascular growing, cell differentiation and hypoxia) are considered as central processes related to preeclampsia [24] and therefore it could support the reliability of the procedure and also the necessity to increase the study of the C8 and C4 genes.

Future combination of centrality indexes and specific clusters selection together with machine learning procedures or genetic algorithm optimization based on groups differentiability or external prediction subset, could reduce even more the final gene space and we are currently working in this direction.

### Diseases and pathway analysis

We manually evaluate through scientific literature the connection of PE with the identified metabolic pathways and diseases. The relationship between preeclampsia and inflammatory, immunologic, angiogenic and hemodynamic responses are very well documented [22-28] and therefore are expected not only in the gene space but also in the metabolic pathways analysis. The metabolic pathway analysis (Table 3) indicates a strong significance of the cancer pathway that is consistent with the disease analysis (Table 2) and also with the VEGF signaling, that is present in several related processes like cytokine-cytokine receptor, angiogenesis and also cancer pathway. With the procedure carried out in the present study a simplification of the metabolic pathways was possible, however, more needs to be done in this area in order to better integrate not only the hubs genes but also the comprehensive data created by the PPI interaction.

Considering the associated metabolic pathways we can notice that several signaling pathways are statistically significant and some of them are connected with PIK3CB, LYN and linked in several cases with TNF (Figure 3) that is a central gene affecting several processes and also widely studied in its relation with preeclampsia [28]. Similarly, SH2B2 that encodes an adapter protein of several tyrosine kinase receptors is also connected with metabolic pathways indirectly related with apoptosis. Contrasting PDIA3 and NDRG1 are not present in the Figure 3 connected with any metabolic pathway, but the recent relation found between PDIA3 and IL11 could open a relationship with cytokine-cytokine receptors [19], specifically through the hematopoietins receptor family. We can also notice (Figure 3 and Table 3) that in the pathways associated with central proteins (the hubs), those highly connected are the cytokine-cytokine receptors, focal adhesion and apoptosis pathways and they contain almost the complete space of genes mainly studied in preeclampsia.

The elevated number of signaling pathways that we found statistically significant, together with the hubs distribution detected in Table 1, seems to point out the idea of a signaling related disease similar to cancer, however, the apoptosis and angiogenic mechanism had been related previously with PE and are also highly represented in our study.

On the other hand, the relationship between PE and cardiovascular diseases is well known. Women with previous history of PE or even pregnancy hypertension present an increased risk of future development of cardiovascular disease or hypertension [29-31]. This is clearly expressed in our results (Table 2). Moreover, our results also suggest a significant relationship between cancer and PE (at this point it is not possible to say if this correlation is positive or negative) indicated by diseases enrichment analysis and also by the metabolic pathways. However, the experimental and epidemiological evidence that could support the influence of PE in future cancer development is for now inconclusive, even when could be reasonably expected by the wide kind of genes that are actually shared between both diseases (i.e. angiogenesis related genes). Several articles report a reduced risk of future cancer development in women with previous history of PE, but others could not find any statistical relationship [32-35].

There are neither clinical signs in common between PE and Alzheimer and nor epidemiological studies. However, the connection between both diseases has been questioned before (at least in late-onset Alzheimer's disease) [36] and was significant in the present study. Therefore, although it is not possible to validate our findings experimentally with the current information, it actually opens new possibilities for future works. A similar problem concerns to other ageing related factors like atherosclerosis, arthritis or longevity (that for obvious reasons will be difficult to explore in PE during long-term studies).

The present study points out several advantages of the bioinformatics analysis but some limitations were also found. The detection of genes that are very well known to be involved in PE thought the applied methodology as well as the consistence of the results across metabolic information could support the novel candidates found, however, it is necessary to reduce even more the genes space applying other methodologies as well as to design new experimental experiences. On the other hand, the limitation of the human protein interaction information suggests that also orthologous genes should be needed in order to increase the PPI covering of the initial dataset and to increase the capabilities of the metabolic pathways and disease enrichment analysis.

## Conclusions

In the present study we applied several bioinformatics tools in order to create a comprehensive initial database of genes statistically related to preeclampsia and a further expansion through the construction of related protein-protein interaction network. Using several centrality indexes for hubs detection, some well know preeclampsia related genes like FLT1, TNF, VEGFA and PGF were detected as well as other genes with high scores, like PDIA3, NDRG1, TBK1, LYN, IQGAP1, DNM1, SAT1, MEN1 and SH2B2 that have been poorly explored or unknown in the current state of the art of preeclampsia physiopathology.

Through disease enrichment analysis we corroborated the well know connection between PE and cardiovascular disease, but we also found a possible link of PE with aging and cancer. Moreover we also found the cancer pathway, focal adhesion, ECM-receptor interaction, apoptosis and cytokine-cytokine receptors interactions metabolic pathways highly represented by the PPI network that are in agreement with some of the preeclampsia-diseases relationship found, as well as with the central topics of the current preeclampsia investigations.

## Methods

### Dataset construction

Experimental proteomic/genomic data comparing normal (N) and preeclamptic (PE) pregnancies was obtained analysing the Gene Expression Omnibus (GEO) database [37]. The datasets considered are represented in Table 4.

**Table 4:** GEO data used for dataset construction

**GEO**	**Sample**	**Method**	**Collection (Weeks)**	**Tissue**	**Ref.**
GSE6573	10(PE), 10(N)	Affymetrix	At delivery	Placenta	[25]
GSE4707^1^	10(PE), 4(N)	Agilent	See notes	Placenta	[6]
GSE25906	23(PE), 37(N)	Illumina	27-38	Placenta	[26]
GSE14722^2^	12(PE), 11(N)	Affymetrix	See notes	Placenta	[27]
GSE10588	17(PE), 26(N)	ABI Human	At delivery	Placenta	[38]

The Table shown the GEO data sources, references and complementary information used in the study.

Notes: 1) The 10 samples were collected for placenta biopsy during delivery. Five of the preeclamptic samples were collected before 31 weeks of gestation and the rest after this time. 2) The 23 samples were collected for placenta biopsy during delivery, however, because in GEO appear two platform types, both were analysed.

Each experiment was analyzed independently in order to reduce the number of genes. In our case we considered an adjusted p-value ≤0.05 and a fold expression ≥2 as discussed elsewhere [6,7,25-27,38,39]. Initially the p-value was obtained by a bootstrapping procedure with 1000 or 10000 iterations (depending on size of the sample) obtaining 645 statistically significant modulated genes, however, applying the false discovery rate (FDR) correction by the Benajmini-Hochberg method [40], this sample was reduced to 330 genes.

In addition, several text mining exploration tools were used to complement the GEO results. There are several tools to perform a text data mining analysis but several of them require extra information (i.e. chromosome region) instead phenotype or diseases notation (i.e. diseases name or related keywords). In our case we choose those methods that do not require previous genetic knowledge of the disease [8]. Moreover, the text mining procedures usually could provide several false positive associations and therefore those tools which also combine text-mining with other data sources in the analysis are preferred [8,41]. Considering these aspects, we used the following tools: PolySearch [42], Candid [43] and Phenopred [44]. Candid and PhenoPred use several heterogeneous data sources to overcome bias while in PolySearch analyse was restricted to PubMed publications. Obviously many other algorithms could be used in alternative. In order to reduce the risk of including biased relationships, the top 10–20 genes/proteins with the highest scores were selected and individually analyzed considering the preeclampsia related scientific publications. Some of the top genes were also present in the previous dataset (GEO), therefore, the final dataset contained 347 genes.

### Protein-protein interaction network (PPI)

The proteins associated with the previous 347 genes were identified and cross-referenced with the IRefIndex (v1.16) [45] and a signaling curated databases [46] that were used to create the protein-protein interaction (PPI) network. The IRefIndex database provide an index of protein interactions available in several databases like: BIND, BioGRID, DIP, HPRD that simplify the task consuming process of inter-database mapping and lead to a comprehensive covering of the available known protein interactions space. On the other hand, this PPI database is easily integrated in Cytoscape. Additionally, many diseases are related with signaling pathways modifications and therefore the inclusion of this interaction database considerable improve the PPI space. The interaction search was restricted to *Homo Sapiens* and includes all kind of experimental procedure as well as some predictive interactions (mainly from the OPHID database). The curation of the final database was performed both, manually and using home-made software to remove duplicate interactions and unify isoforms notation with unique genes. We obtained our final PPI network with 3279 interactions and 2400 nodes.

Some of the proteins present in our initial dataset had not any known experimental interaction (at least in humans) and therefore the 2400 nodes cover only 234 (67.45%) genes of the initial set (347). The network visualization and network topology indexes, calculated in the hubs detection process, were carried out using Cytoscape 2.8.2 and CytoHubba [47,48].

Several methodologies are available for hubs and essential genes identification, and all of them with the respective advantage and limitations [47,49-54]. Some strategies are the use of genetic algorithm or machine learning procedures [49,50], however, the centrality approaches are by far the most applied procedures even by simplicity and because several studies had being pointed out its applicability [47,51,52]. Therefore, several centrality indexes were evaluated: Betweenness, bottleneck, density of maximum neighborhood component (DMNC), node degree, edge percolation component (EPC), eccentricity, maximal clique centrality (MCC), maximum neighborhood component (MNC), radiality and stress [47]. On the other hand, to obtain a scoring index we created the measurement (*Score I*) as follow:

(1) $$\text{Score}I=\sum_{i=1}^{Nc}\left[\left(1-\frac{\max\left(Ic_{i}\right)-Ic_{i}}{\max\left(Ic_{i}\right)-\min\left(Ic_{i}\right)}\right)\cdot 100\right]$$

Where Ic_i_ is the values of centrality indexes and i = 1…Nc, and is the number of calculated centrality indexes (Nc = 10). As we can note, *score I* is the sum of all the indexes percent value after individual normalization and therefore is restricted to a maximal value of 100×Nc which simplify even better the top genes selection. With the normalized centrality indexes we also performed a model-based clustering analysis using R-package [53] in order to study hubs distribution with respect to centrality ranks. We also performed a communality (or cliques) network analysis by clique percolation method using CFinder [54]. The communality analysis provides a better topology description of the network including the location of highly connected sub-graphs (cliques) and/or overlapping modules that usually correspond with relevant biological information.

### Pathway and diseases enrichment analysis

The pathways and diseases enrichment analysis were performed through the DAVID bioinformatics resource 6.7 [55], exploring the well know databases: KEGG, BioCarta and Reactome (pathways related) as well as OMIN and Genetic Association Database (GAD) (diseases related analysis). This online resource (DAVID) integrate, in a faster computational analysis, a wide range of enrichment analysis thought different databases providing also a substantial statistical description. The analysis was carried out considering the complete gene space of the PPI network. We also used DAVID in order to perform a gene ontology enrichment analysis in the obtained clusters.

## Competing interests

The authors declare that they have no competing interests.

## Authors’ contributions

ET implemented the methods and worked out in the database processing. ET, JB and IR designed the study as well as worked out in the biological background for the discussions. All authors contributed to the writing of the manuscript and read and approved the manuscript.
